# Mapping the Neural Basis of Wake Onset Regularity and Its Effects on Sleep Quality and Positive Affect

**DOI:** 10.3390/clockssleep7010015

**Published:** 2025-03-13

**Authors:** David Negelspach, Kathryn E. R. Kennedy, Alisa Huskey, Jungwon Cha, Anna Alkozei, William D. S. Killgore

**Affiliations:** Department of Psychiatry, University of Arizona, Tucson, AZ 85724, USA

**Keywords:** actigraphy, circadian rhythms, sleep, default mode network, mood disorders, neuroimaging

## Abstract

The regularity of sleep–wake cycles is a defining characteristic of normative sleep patterns that are typically associated with proper circadian rhythmicity. The previous literature indicates that consistent patterns of sleep and wake are associated with improved sleep quality and cognitive functioning. Conversely, sleep irregularity has been associated with reduced well-being and inefficiency in resting-state neural networks. This study investigated the relationship between specific sleep regularity measures and outcomes, including emotional affect, sleep quality, and resting-state functional connectivity. We found that variability in wake onset predicted poorer sleep quality and reduced positive affect. Furthermore, sleep regularity measures were associated with altered functional connectivity between the posterior cingulate cortex and regions involved in emotional processing. We propose that alterations in default mode network (DMN) connectivity linked to sleep irregularity reflect disruptions in emotional processing and sleep quality.

## 1. Introduction

The proper maintenance of sleep–wake rhythms plays a crucial role in optimal brain health and cognitive functioning. Sleep serves key restorative functions, including facilitating metabolic clearance [1], modulating synaptic strength [2,3,4], and supporting memory consolidation [5,6,7,8]. Failure to meet biological sleep needs diminishes these processes and can, in turn, lead to significant impairments in cognitive functions, including attention [9] and working memory [10], and poses an increased risk for neurodegenerative diseases [11,12]. Recent evidence suggests that vital neurobiological processes that occur during sleep not only depend on adequate durations but on the regularity of sleep episodes as well. Understanding how the regularity of sleep–wake rhythms is associated with functional changes in the brain may provide insight into the behavioral outcomes associated with irregular sleep patterns.

When discussing the importance of sleep regularity, it is helpful to first acknowledge the most established metric of preventative sleep medicine: adequate duration. Many large meta-analyses show that failure to consistently obtain the recommended 7 to 9 h of sleep per night (for adults) [13] is linked to an increased risk of a range of disorders, including cardiometabolic diseases: heart disease [14,15,16], obesity [17], and diabetes [18,19] (for reviews, see [20,21,22,23]). Many of these same conditions also show a similar trend with respect to sleep regularity, whereby irregular sleep rhythms are associated with an increased risk of adverse outcomes [24,25,26]. A recent study reported that sleep regularity, as compared to sleep duration, was a stronger predictor of all-cause mortality [27]. To be clear, this comparison was not carried out to downplay the importance of maintaining a healthy sleep duration, as this can pose an immediate and consequential impact on survival [28]. The purpose of this comparison is to emphasize the importance of sleep regularity, as evidence suggests that in the case of normative sleep patterns, regularity of sleep, as opposed to sleep duration, may have a more long-term impact on longevity.

In addition to being linked with the risk of morbidity and mortality, evidence suggests that sleep regularity is also important for cognitive performance [29]. Studies have demonstrated that individuals with more consistent sleep and wake times score higher on measures of alertness and executive function [30,31]. This effect extends beyond laboratory settings, as sleep regularity has been shown to predict academic performance in college students [32,33], and cognitive functioning in the context of age-related cognitive decline and beta-amyloid pathologies [34,35,36,37]. These findings suggest that the cognitive benefits of sleep are not only a matter of quantity but also consistency, highlighting the crucial role of regular sleep patterns in maintaining optimal brain health across the lifespan. It should also be noted that several of these studies reported such findings while controlling for variance in sleep duration. This is an important consideration, as sleep loss can be a strong predictor of neurocognitive deficits as well [38,39].

Beyond physical health and cognitive functioning, sleep regularity also plays a vital role in emotional well-being [40,41], whereby individuals with more irregular sleep patterns also score higher on reported depression and anxiety symptoms [42,43,44,45]. This may be particularly problematic for vulnerable populations, such as those with post-traumatic stress disorder (PTSD), where sleep irregularity moderates the link between wake-after-sleep onset (WASO) and depressive symptoms [46]. In this case, consistent sleep–wake timing appears to act as a protective mechanism between sleep fragmentation and subsequent depression severity. These data provide insight into how regularity of sleep–wake timing may prevent the risk of mood disturbances and improve general well-being.

Sleep regularity relies on coordinated regulation between cortical and sub-cortical systems. One network that may have direct relevance for sleep onset and regularity is the default mode network. Comprising the posterior cingulate, bilateral inferior parietal lobules, and ventromedial prefrontal cortex [47], this network is primarily dominant at rest and shows coordinated functional activity. Cognitive functions associated with the default mode network (DMN) include self-referential thought, autobiographical memory, and mind-wandering (for a review, see [48]). As the primary network that manages our internal narratives, its function can either promote positive feelings of self-worth or negative ruminations [49,50,51]. What makes this network particularly relevant to sleep is that this resting mind state is likely the cognitive state experienced prior to the transition into the initial stages of sleep.

Neuroimaging studies have established a clear link between DMN functionality and sleep. Functional connectivity between specific DMN nodes and cortical regions has been shown to predict polysomnographic measures, including sleep efficiency, total sleep time, and time spent in REM sleep [52]. Additionally, insomnia symptoms have been associated with decreased connectivity within key DMN nodes [53]. This relationship is theorized to stem from the DMN’s role in regulating mental states: heightened DMN activity may promote rumination, which in turn corresponds to sleep disturbances [54,55]. These findings also extend to sleep regularity specifically, as the efficiency of DMN functional connectivity correlates positively with the sleep regularity index [56]. These studies suggest that DMN has an impact on sleep-related arousal, as well as the long-term maintenance of sleep/wake rhythm timing.

Altered DMN connectivity may also serve as a critical neurobiological mechanism linking irregular sleep patterns to impaired well-being. As the network responsible for managing internal narratives, the DMN can promote either positive feelings of self-worth or negative ruminations [49,50,51]. This is evident in psychiatric conditions like major depressive disorder, where key DMN nodes exhibit disrupted cortical functional connectivity [57]. The high co-morbidity between depression and sleep disturbances [58] raises the possibility that the altered connectivity of the DMN may be involved. Together, this highlights the DMN as a target for understanding and addressing sleep-related emotional dysregulation.

While existing research underscores the critical role of sleep regularity in cognitive functioning and emotional well-being, key questions remain regarding which aspects of regularity drive these outcomes and whether the DMN plays a central role in this process. Several methods exist to quantify sleep regularity, but it remains unclear which specific factors—such as sleep onset or sleep offset—are the most reliable predictors of emotional affect and sleep quality. Additional research is needed to pinpoint the differential effects of specific sleep regularity measures while controlling for sleep duration. Moreover, it is unclear what patterns of abnormal connectivity within the DMN emerge as a result of irregular sleep patterns. Understanding which functional regions display abnormal connectivity would provide evidence of potential mechanisms underlying sleep irregularity, emotional affect, and sleep quality. To investigate this, a sample of healthy adults underwent resting-state functional magnetic resonance imaging (fMRI). The sample consisted of individuals without apparent signs of circadian dysregulation who also regularly slept 7–9 h a night. We hypothesized that variability in sleep and wake times would disrupt the functional connectivity of the DMN and correlate with greater self-reported depressive symptoms.

## 2. Results

The demographics of all participants included in the study are provided in Table 1. Descriptive statistics for the actigraphy and self-reported metrics related to sleep–wake onset variability and sleep maintenance are presented in Table 2. These metrics include wake onset time, sleep onset time, PSQI sleep efficiency, sleep quality, and self-reported positive affect. Sleep regularity was measured separately by the standard deviation in sleep and wake onsets, as these factors may independently contribute to the variance in outcome variables, each having its own unique association with behavioral and neural outcomes. To explore this possibility, both measures were separately included in subsequent multivariate modeling and functional connectivity analyses to compare significant outcomes.

Prior to model specification, a partial correlational matrix was constructed to detect whether changes in regularity of wake vs. sleep time were individually associated with sleep duration. This approach serves to elucidate the unique relationships between separate measures of sleep regularity while controlling for potential confounding factors. We found no significant association between the total sleep time (TST) and wake onset variability (WOV) (R_WOV.TST_ = 0.22; *p* = 0.41) or sleep onset variability (SOV) (R_SOV.TST_ = –0.28; *p* = 0.30). There was a significant partial correlational coefficient between SOV and WOV (R_SOV.WOV_ = 0.61, *p* = 0.01) while controlling for TST. This result illustrates that individuals in our sample who experienced more variability in their sleep–wake timing were not necessarily experiencing a relative decrement in TST.

A multivariate regression analysis was conducted to calculate the association between sleep regularity and outcome measures of sleep quality and positive emotional affect. The average sleep duration was included as a covariate to control for a key confounding variable that may influence these outcomes. A combination of leverage and Cook’s distance was used to calculate each observation’s influence on outcome variables in the regression model to accurately identify significant outliers. Observations with Cook’s distance exceeding 1 were considered influential outliers and removed from the regression model [59]. Only one such outlier was identified and removed. The multivariate regression model revealed that wake onset variability was a significant predictor of the SQS score (b = 0.35, β = 0.69, *p* < 0.01) and positive affect (b = –0.28, β = –0.55, *p* < 0.03). The sleep quality scale has an inverse relationship with sleep quality, meaning higher scores reflect worse sleep quality. Thus, this regression model indicates that increased WOV is associated with impaired sleep quality and reduced positive affect. These results remain significant after the Benjamini–Hochberg correction for false discovery. The regression model for WOV was robust relative to the inclusion of subjective sleepiness (*p* < 0.04) as a covariate [60].

**Figure 1:**
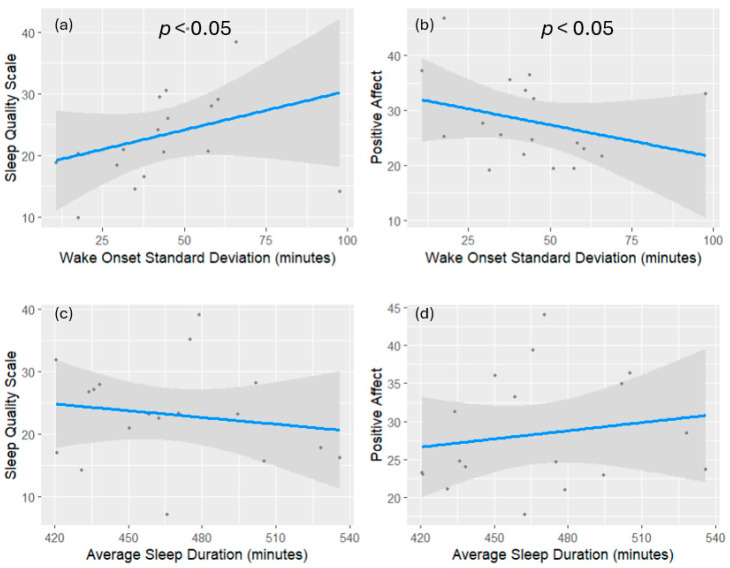
Visualization of each individual predictor’s association with the dependent variable of interest. Panels (**a**,**b**) show associations between wake onset variability, sleep quality (**a**), and positive affect (**b**). Panels (**c**,**d**) show the associations between average TST, sleep quality (**c**), and positive affect (**d**). The sleep quality scale is scored inversely, meaning a higher score on the SQS represents worse sleep quality. The grey shaded areas represent the 95% confidence intervals of each regression. The type II MANOVA test of the multivariate regression model confirms that sleep regularity significantly affected both subjective sleep quality and emotional affect while controlling for TST: Pillai’s Trace, Wilks’ Lambda, and Hotelling–Lawley trace all yield significant results (*p* < 0.0099).

To compare individual measures of sleep regularity and potential unique associations with outcome measures, a subsequent multivariate regression model was calculated using SOV. The results showed that SOV is not a significant predictor of either sleep quality or positive affect. Sleep durations within a 7–9 h range did not significantly predict either outcome (Figure 1).

**Figure 2:**
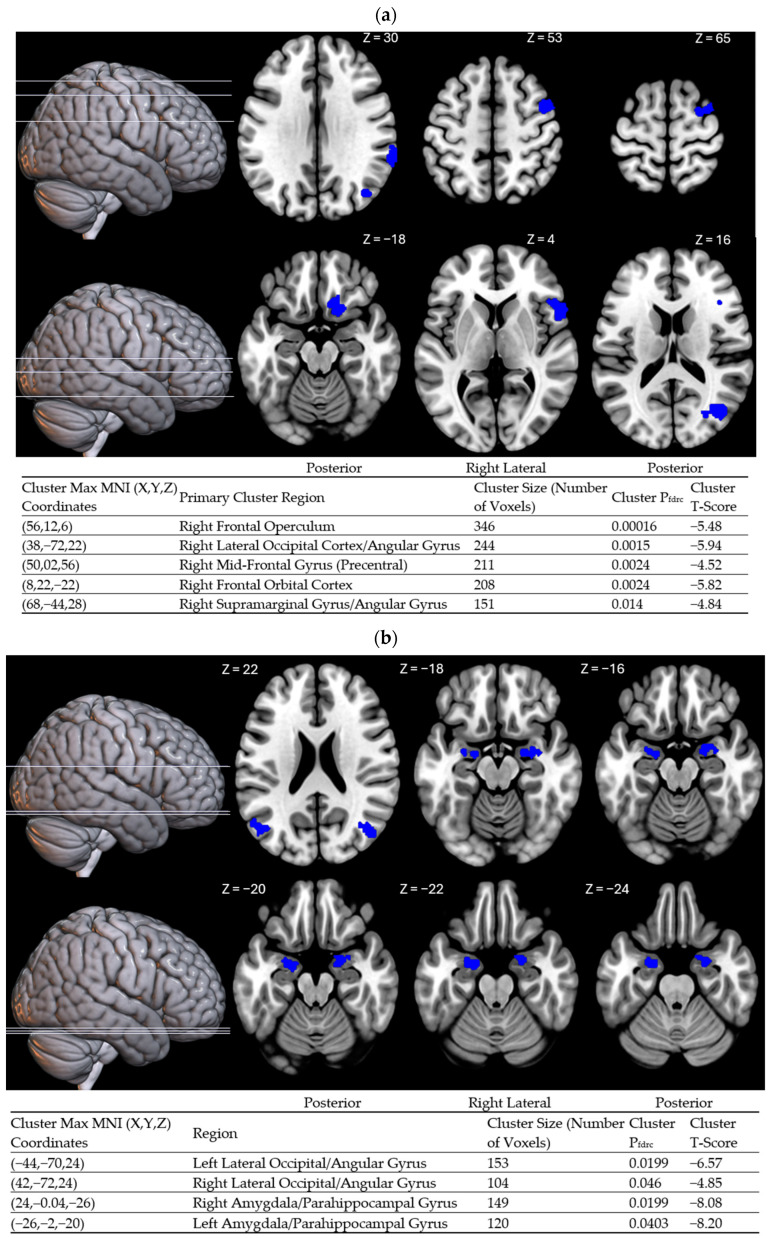
Negative functional connectivity with the posterior cingulate cortex at rest in association with sleep regularity. MNI coordinates reported the location of the maximum voxel signal within the significant cluster. The regions shown in blue represent areas with significantly reduced functional connectivity with the posterior cingulate cortex, whereas areas shown in red represent positive functional connectivity. (**a**) shows wake onset variability as the primary covariate of interest, whereas (**b**) shows sleep onset variability as the primary covariate of interest. Age, gender, and sleep duration on the day prior to functional scans were included as covariates of no interest. The individual voxel threshold was set at *p* < 0.005. All clusters shown are significant at a false discovery rate correction of p_fdrc_ < 0.05. Anatomical regions were reported using MNI coordinates of cluster max voxels cross-referenced with the automatic anatomical labeling atlas [63].

To further probe the potential effects of sleep-interval irregularity on waking cognitive states, a functional connectivity analysis was conducted using the posterior cingulate as the primary seed region of the DMN. This region was chosen because it is a primary driver of self-focused mentation [61,62]. A covariate of interest analysis was conducted on WOV, which revealed heightened negative functional connectivity between the posterior cingulate and several cortical regions with greater WOV (Figure 2a). Areas showing potentiated negative functional connectivity with greater WOV were lateralized in the right hemisphere and include the supramarginal gyrus (BA 40), angular gyrus (BA 39), mid-occipital cortex (BA 19), frontal orbital cortex (BA 11), pre-central gyrus/mid-frontotemporal gyrus (BA 6), and frontal inferior operculum (BA 38). A post hoc rank order correlation revealed a significant association between altered functional connectivity in the angular gyrus and composite score on the PANAS (ρ = –0.47, *p* = 0.038).

## 3. Discussion

The results generated from this study demonstrate the impact of sleep regularity on functional connectivity and behavioral outcomes in an otherwise healthy population. Variability in wake onset predicted worse sleep quality and reduced positive affect. Notably, these effects were found after controlling for sleep duration: Direct partial correlations indicated that neither variability in wake onset nor sleep onset was associated with changes in sleep duration within the recommended 7–9 h range. This supports the conclusion that the observed effects associated with sleep regularity are not confounded by differences in overall sleep duration.

Comparing findings within the sleep regularity literature is complicated by the fact that irregularity is defined in numerous ways (i.e., social rhythm regularity, sleep duration consistency, inter-daily variability, sleep midpoint, etc.). Differences between these metrics may each capture unique variances in outcome variables [64]. Our comparison of multivariate models sought to determine whether sleep onset or wake onset variability was a better predictor of the positive outcomes associated with regular sleep. We found that wake onset variability was a stronger predictor of both sleep quality and positive affect, whereas sleep onset variability did not significantly predict either outcome.

This finding aligns with the two-process model, which posits that irregular sleep intervals disrupt circadian rhythmicity (Process C) without necessarily affecting the ability to meet daily sleep needs (Process S) [65,66]. However, it is important to note that these two processes interact, and significant deficits in sleep regularity may eventually impair sleep duration. Given that our sample consisted of healthy individuals meeting strict criteria to exclude other factors that could influence circadian regulation, we conclude that, among those obtaining the recommended amount of sleep, maintaining a more regular wake time is positively associated with both sleep quality and positive affect.

One possible explanation for this finding is that wake onset showed greater variability within our sample, enhancing the detection of its relationship with these outcomes. Alternatively, the timing of light exposure in the circadian advance zone may play a more significant role in photoentrainment stability. Light exposure following wake onset is more likely to include natural morning light, which induces a stronger phase response than artificial light experienced prior to sleep [67]. Frequent disruptions in the timing of morning light exposure may impair stable photoentrainment, misaligning the optimal circadian timing for sleep with actual sleep periods. Circadian misalignment has been shown to alter sleep architecture [68,69,70]. REM sleep, in particular, is strongly influenced by the circadian pacemaker [71], and it is proposed to have a role in emotional processing [72] and next-day affect [73,74]. Therefore, the influence of circadian timing on emotional processing during sleep provides one potential mechanism linking WOV with positive mood.

In the context of sleep regularity, another possible mechanism linking WOV with positive, but not negative, affect is the circadian modulation of dopaminergic activity. Recent evidence in mice suggests that dopaminergic activity has a bidirectional association with circadian/infradian rhythms, particularly within the mesolimbic pathway [75]. This pathway is crucial for reward processing and positive mood states. Given the potential for the regularity of behavioral schedules to affect the circadian amplitude [76], this may extend to dopaminergic rhythms in the limbic system, selectively impacting positive affect while leaving negative affect relatively unchanged. Future research could benefit from studying circadian differences correlating with personality measures that are closely linked to the dopaminergic regulation of mood.

Prior research has shown that the efficiency of intrinsic default mode network (DMN) connectivity positively correlates with sleep regularity [56]. Building on these findings, we investigated which areas show impaired connectivity with the PCC both within and outside of the DMN. Consistent with previous studies, greater wake onset variability (WOV) was linked to reduced connectivity between the PCC and the angular gyrus, which are both core DMN nodes. Additionally, high WOV was associated with decreased connectivity between the PCC and the inferior frontal gyrus, orbitofrontal cortex, and supramarginal gyrus. This pattern suggests that irregular wake timing may destabilize the functional capacity of the DMN to coordinate with areas supporting adaptive decision making [77,78,79] and emotional stability [80]. As such, the DMN may play a role in linking irregular sleep patterns to impaired cognitive abilities.

To further investigate the effects of sleep regularity, we conducted a second functional connectivity analysis correlating PCC connectivity with sleep onset variability (SOV). While no significant correlations were found between SOV and self-reported measures, the possibility remained that it might be linked to functional deficits. This hypothesis was confirmed: Sleep onset variability was associated with reduced connectivity between the PCC and clusters in the temporal lobe, including the amygdala, hippocampus, and parahippocampal gyrus. Considering that the amygdala plays a major role emotional processing [81] and the hippocampus plays a major role in memory encoding [82], we propose that impaired connectivity with the PCC and these regions may disrupt the internal processing of emotional memories. A similar pattern was observed in a neuroimaging study of postpartum depression, where women with this condition exhibited impaired PCC–amygdala connectivity compared to non-depressed mothers [83]. This evidence further supports our hypothesis that sleep irregularity is linked to dysregulated emotional processes involving the DMN.

The concept of sleep regularity, as it relates to health and cognitive functioning, is closely related to a well-established interaction between circadian rhythmicity and its role in regulating sleep-related processes. This interaction is central to theories such as the two-process model and the social zeitgeber theory [84,85], which predict that optimal sleep occurs when sleep needs and the circadian phase overlap synergistically. As such, regularity in sleep/wake rhythms likely facilitates stable photoentrainment corresponding to sleep intervals. This study expands on this body of literature by identifying specific functional abnormalities of the posterior cingulate associated with specific sleep regularity measures. The regions showing altered functional connectivity with the PCC are involved with cognitive functions that are closely related to behavioral deficits associated with sleep irregularity. Furthermore, this study highlights how different measures of sleep regularity have distinct functional and behavioral correlates. Sleep onset and wake onset differ in their ability to predict sleep quality and positive affect. These variability measures also have unique functional associations with DMN connectivity. Sleep onset variability may have a less noticeable effect on subjective reports but a stronger relationship with functional connectivity in brain regions involved in emotional processing. While both are measures of sleep regularity, this could be an indication of separate underlying cognitive functions associated with wake onset, versus sleep onset. However, this could also be confounded by unequal variance in the study sample, as young adults who participated displayed higher variability in wake timing. As both are plausible explanations, future research would benefit from investigating whether sleep regularity demonstrates unequal variance between sleep and wake onsets and whether potential differences in their behavioral/mood-related correlates.

## 4. Strengths and Limitations

This study has several strengths and weaknesses that contribute to the robustness of its findings. First, a major strength of this study is the rigorous quality control actigraphy data were subjected to, ensuring accurate sleep–wake estimates. Sleep metrics were double-rated by independent scorers, cross-referenced with self-reported sleep and wake times, and supplemented by light sensor data for greater precision. Another strength is the methodological effort to minimize the confounding effects of circadian arousal on resting-state cognition. To control for this, MRI scans were conducted within the same time window for all participants. Finally, to avoid bias in the regression modeling, sleep regularity was measured using a raw variability metric, calculated separately for sleep onset and wake onset times. This approach eliminates assumptions about shared variance between the two measures of sleep regularity.

The study also has limitations that should be acknowledged. One key limitation is the small sample size, which may limit the generalizability of the findings and reduce statistical power. Additionally, due to recruitment practices, participants were primarily from an undergraduate population and primarily white, which may not accurately reflect the demographics of the general population. Another consideration is the fallibility of subjective measures, which are vulnerable to response bias and may potentially introduce inaccuracies in the data. Furthermore, while measures were taken to address incomplete actigraphy data from two participants (e.g., sensitivity analyses to ensure robustness), this remains a limitation that could influence the final results. Finally, resting-state functional connectivity is inherently subject to uncertainty regarding the participants’ cognitive states during the scan. Individual differences in resting-state cognitive habits may introduce variability, complicating the interpretation of changes in resting-state network functionality across participants.

Another limitation of this study is the duration of actigraphy monitoring. Longer recording periods are recommended for a more accurate assessment of intrinsic circadian rhythmicity. A seven-day period was chosen to balance data quality with participant burden and compliance. Future studies would benefit from extended actigraphy monitoring to enhance the reliability of sleep regularity measures.

Despite these limitations, the multiple quality control measures and standardized procedures strengthen the validity of the results, offering valuable insights into the relationship between sleep regularity, associated changes in self-reported measures, and trends in resting-state functional connectivity with the DMN.

## 5. Materials and Methods

A cohort of 18 individuals (18–35 years; 13 females) were recruited locally from the Tucson, AZ region. Participant screening was conducted online and in a follow-up phone interview. Both relied on the self-report of the participant to provide true and accurate answers. The inclusion criteria required they claim to be free of any neurological conditions, have no history of current or past drug use, and are not currently taking any sleep medications. Additional criteria required that they be proficient in English and be right-handed, which was confirmed upon visiting the lab in person. Provided that these criteria were met, participants were provided with an actigraphy watch (Actiwatch; Philips Respironics, Murrysville, PA, USA) and instructed to maintain their typical lifestyle/behavior and sleep–wake schedules. This included adherence to their typical work/academic schedules. Actigraphy was collected for one week before the MRI scan. Digital sleep diaries were also completed each morning. Functional MRI visit dates were provided ahead of time to allow adequate time to schedule time off or confirm the absence with their academic institution.

Following a week of home monitoring, participants visited the laboratory for a neuroimaging assessment. Beginning at 10:30 am, participants completed a battery of behavioral assessments, including the Positive Affect Negative and Affect Schedule (PANAS) [86], Pittsburg Sleep Quality Index (PSQI) [87], sleep quality scale (SQS) [88], and Karolinska Sleepiness Scale (KSS) [89]. Once completed, participants were escorted to the 3-Tesla Siemens MAGNETOM Skyra scanner, fitted with earplugs, and laid supine in a comfortable position. A Siemens MAGNETOM Skyra 3T MRI scanner (Erlangen, Germany) was used for all data acquisition. A 32-channel head coil with a display mirror was positioned towards a projector screen displaying a fixation point. The scan sequence began with a T1-weighted MPRAGE, followed by field mapping. As part of the Siemens scanning protocol, dummy scans were performed prior to data collection. Resting-state functional scans were then acquired using a T2-weighted echo-planar imaging (EPI) sequence with the following parameters: TR = 2000 ms, TE = 36 ms, flip angle = 90°, slice thickness = 2 mm, in-plane resolution = 2 × 2 mm, matrix = 120 × 60, and field of view = 240 mm. Sixty slices were acquired per volume in an interleaved order, covering the entire brain. The phase encoding direction was anterior-to-posterior (A >> P). Echo spacing was 0.71 ms, and bandwidth was 1602 Hz/Px. The in-plane acceleration factor was set to 2 (GRAPPA). A total of 360 volumes were collected during each resting-state scan for a total acquisition time of 12 min. Participants were instructed to keep their eyes open, fixate on a crosshair displayed on a screen visible through the mirror, and let their minds wander. Motion correction was applied during acquisition to minimize movement-related artifacts. Additional motion corrected was applied during preprocessing described below.

Functional and anatomical images were pre-processed in the SPM12 toolbox CONN version 22a according to the default preprocessing pipeline [90,91,92,93]. This consisted of realignment with distortion correction (unwarping) [94], slice timing correction of interleaved slices, conservative outlier detection settings (global signal 3σ above mean or movement threshold of 0.5 mm) [95,96], skull stripping, tissue segmentation, and normalization to the MNI space based on the default IXI-549 tissue probability map template [97,98,99,100]. Functional images were then smoothed using a Gaussian kernel full-width half maximum (FWHM) of 6 mm. Individual seed-based connectivity (SBC) maps were created using the network-level and anatomical regions of interest [101]. As part of the required CONN default analysis pipeline, additional denoising variables were created based on segmented tissue time series. Denoising and movement variables as determined via the “aCompCor” methodology were included later as covariates in second-level analyses [102,103]. This includes nuisance covariates of cerebrospinal fluid, white matter, and motion parameters.

The strength of functional connectivity was determined by Fisher-transformed bivariate correlation coefficients from a weighted general linear model defined separately for each pair of seed and target areas. This effectively models the association between the BOLD signal time series across regions. Group-level analyses were performed using a general linear model (GLM) [90]. For each individual voxel, a separate GLM was estimated, with first-level connectivity measures at this voxel as dependent variables and covariates as independent variables. Voxel-level hypotheses were evaluated using multivariate parametric statistics with random effects across subjects and sample covariance estimation across multiple measurements. Inferences were performed at the level of individual clusters (groups of contiguous voxels). Cluster-level inferences were based on parametric statistics from Gaussian Random Field theory [90,104]. The results were thresholded using a cluster-level false discovery rate correction of p_fdrc_ < 0.05.

Actogram scoring and assessments of sleep regularity measures were conducted using a multi-step process to ensure accuracy. First, sleep intervals were determined using the built-in Actiware algorithm (Philips Respironics). Next, two separate lab technicians who were trained in actigraphy scoring made their own determinations for the assessment of sleep intervals. These assessments also involved light-spectrum data captured by actigraphy devices to corroborate activity and help raters make a more informed decision. Actigraphy devices also provided data specifying if the device was “off-wrist”, which was also used to inform sleep interval scoring. Finally, rater scores were cross-referenced with subjective sleep markers and sleep diaries. Final determinations for sleep regularity measures were made using the time-point that showed the most overlap between this combination of factors and intervals.

To target a healthy population free of any dysregulated circadian sleep–wake patterns, only participants that met the following requirements were included in further analyses: sleep efficiency exceeding 80 percent, average sleep duration between 7 and 9 h, sleep intervals occurring within a 9:00 PM–10:00 AM time frame, no light exposure during sleep intervals interval lasting more than 1 h or brighter than 1000 μW/cm^2^ (determined by light-sensor data equipped on Actiwatch), and continuous wear. Two participants did not meet this last requirement and were found to have insufficient actigraphy data (<60%). In these cases, subjective sleep markers, sleep diary entries, and light data were utilized to approximate sleep intervals. This approach ensured that all participants contributed to the analysis despite the data gaps identified in their actograms. To assess the robustness of our findings, all analyses were conducted both with and without the inclusion of these two participants. Importantly, excluding these participants did not alter the outcomes of the analysis, indicating that their inclusion did not significantly impact the results reported in this study.

The protocol for this study was reviewed and approved by the University of Arizona Institutional Review Board (IRB 1907791609) and the US Army Office of Human Research Oversight (OHRO). This study was conducted in accordance with the ethical standards outlined by the University of Arizona’s IRB for the protection of human subjects. All participants provided informed consent before participating in any study procedures. Compensation was provided for their time and effort in accordance with the university’s compensation guidelines. Additionally, participants were assured of their right to withdraw from the study at any time without penalty or consequence. Confidentiality and privacy of all participants were strictly maintained throughout the study.

## Figures and Tables

**Table 1:** Demographic variables for study participants.

Demographics	
Age	Mean = 23, range = [18,19,20,21,22,23,24,25,26,27,28,29,30,31]
Biological Sex	*n* = 18, female = 11
Race/ethnicity	White = 8 Hispanic = 8 African American = 1 Other = 1
Education	High school graduate = 3 Some college = 8 Associate’s degree = 2 Bachelor’s degree = 4 Master’s degree = 1

**Table 2:** Summary statistics of sleep interval variables used for screening and analysis. Reported values for sleep intervals are calculated as the mean of individual weekly means of actigraphy data. Sleep quality scale and positive affect reflect self-reported component scores of the respective questionnaire.

Variable	Mean (σ)
Sleep Onset	11:10 PM (33.44 min)
Wake Onset	06:56 AM (45.17 min)
Sleep Duration	7 h 43 min (35.11 min)
Sleep Efficiency	93.36% (8.59%)
Sleep Quality (SQS)	23.28 (8.16)
Positive Affect (P.A.N.A.S.)	28.22 (7.79)

## Data Availability

Neuroimaging data presented in this article are not shared publicly due to the presence of metadata that can be extracted from raw or processed images, which are classified as protected health information. Data that can be de-identified and contain no personal health information of participants involved may be available upon request from the corresponding author in accordance with HIPPA and other ethical restrictions.
